# The Study of an Optimal Robust Design and Adjustable Ordering Strategies in the HSCM

**DOI:** 10.1155/2015/517245

**Published:** 2015-09-14

**Authors:** Hung-Chang Liao, Yan-Kwang Chen, Ya-huei Wang

**Affiliations:** ^1^Department of Health Services Administration, Chung Shan Medical University, No. 110, Sec. 1, Jian-Koa N. Road, Taichung 402, Taiwan; ^2^Department of Medical Education, Chung Shan Medical University Hospital, No. 110, Sec. 1, Jian-Koa N. Road, Taichung 402, Taiwan; ^3^Department of Distribution Management, National Taichung University of Science and Technology, No. 129, Sanmin Road, Sec. 3, Taichung 402, Taiwan; ^4^Department of Applied Foreign Languages, Chung Shan Medical University, No. 110, Sec. 1, Jian-Koa N. Road, Taichung 402, Taiwan

## Abstract

The purpose of this study was to establish a hospital supply chain management (HSCM) model in which three kinds of drugs in the same class and with the same indications were used in creating an optimal robust design and adjustable ordering strategies to deal with a drug shortage. The main assumption was that although each doctor has his/her own prescription pattern, when there is a shortage of a particular drug, the doctor may choose a similar drug with the same indications as a replacement. Four steps were used to construct and analyze the HSCM model. The computation technology used included a simulation, a neural network (NN), and a genetic algorithm (GA). The mathematical methods of the simulation and the NN were used to construct a relationship between the factor levels and performance, while the GA was used to obtain the optimal combination of factor levels from the NN. A sensitivity analysis was also used to assess the change in the optimal factor levels. Adjustable ordering strategies were also developed to prevent drug shortages.

## 1. Introduction

Recently, one of the most important strategy issues for hospital administrators is to measure the performance of supply chain management (SCM). Bhatnagar and Sohal [1] researched competitiveness among SCMs, including the impact of plant location, uncertainty of supply, and manufacturing practices; they found a significant relationship among these factors. Marucheck et al. [2] studied product safety and security in the global SCM to address certain research problems, including regulation, product lifecycle management, and supplier relationships. Bendavid and Boeck [3] presented a radiofrequency identification (RFID) technique to improve hospital supply chain management (HSCM) in order to improve in turn the end-to-end traceability of medical products.

He and Lai [4] constructed an SCM model in order to identify the relationships between operational and strategic integration. Their results showed that while customer-action-based service has a direct positive effect on strategic integration, operational integration has a direct positive effect on product-based service. Kelle et al. [5] studied a case of an SCM in a pharmacy and its managerial practices in a hospital. They found that the conflicting goals of various stakeholders created a tradeoff effect among the operational, tactical, and strategic levels. They determined that decision-support tools are necessary to facilitate and improve performance in management practices. Ghandforoush and Sen [6] presented a prototype supply chain decision-support system (DSS) for platelet production and the schedule of a blood mobile run by a regional blood center. Their goal was to optimize the delivery of platelets from production centers to hospitals using the supply chain DSS. Their results suggested that applying the supply chain DSS to the platelet production plan and the mobile assignment schedule was a better means of meeting daily demand. Lin et al. [7] investigated the innovation of channel integration and its effect on SCM performance: their results indicated that there is a significant relationship between market orientation and SCM performance and that value cocreation and value constellations are positively associated with SCM performance. Zhang et al. [8] constructed a conceptual model with inventory visibility, which is a critical part of SCM. Their results emphasized that inventory visibility enables companies to make their SCM as effective as possible.

Based on the literature summarized above, most SCM research had paid attention to the product production for suppliers, manufacturers, and customers. There is little research on HSCM. Also, in Taiwan, most of hospitals' purchasing professionals have slight recognition or may not be familiar with the concept of supply chain [9]. Besides, because, according to the medical law, hospitals cannot market medical products, most hospitals do not know how to use the e-health purchasing system, to improve the business performance. Therefore, to establish a HSCM model and to connect the e-health purchasing system with the SCM plan in Taiwan will be an important issue in the hospital management in the future.

In addition, to explore the HSCM's performance, the total system cost (TSC) should also be taken into consideration. Lapierre and Ruiz [10] proposed an innovative approach to schedule inventory activities based on total cost of logistics to improve hospital supply system. The study tested a real hospital case in Montreal, Canada, and found that, with better coordination of purchasing and procurement, the hospital logistic management may be improved. de Vries [11] redesigned the inventory systems in a health care setting and suggested that project managers should understand how to manage inventory in order to reduce costs. Thus, the project managers can assist the decision-making process in hospital inventory systems. Wang et al. [12] proposed a purchasing policy on certain surgical materials in the Chang Gung Memorial Hospital, Taiwan, attempting to construct a model to minimize the total cost of consignment inventory, including a deteriorating item, in the condition that the buyer has warehouse capacity constraint. This model enables managers to understand how to obtain lower holding cost and how to enjoy more flexible cash management. Chakraborty et al. [13] researched the impact of supply chain collaboration and its components in the context of healthcare service sector. The result helped to construct the supply chain collaboration with value cocreation and firm performance.

Along with reducing the TSC, improving the patient safety level (PSL) is also an important issue to enhance high quality care in the performance of HSCM in Taiwan; Liao's research on HSCM [14] investigated how to dispatch the obtaining quantity to minimize total cost while taking patient safety into account. The result showed that a centralized purchasing center is useful to effectively coordinate the hospitals' cost and the PSL. Liao and Chang [15] adopted an optimal approach for parameter settings to establish the adjustable contracting capacity for the hospital supply chain logistics system, using the hospitals in the North Alliance of the Department of Health of Taiwan. The results showed that the proposed optimal approach can effectively predict responses, the TSC, and the PSL. Uthayakumar and Priyan [16] made research to optimize SCM for a pharmaceutical company and a hospital in order to improve health policy, public health, patient safety, and strategic decision-making. Liao et al. [17] developed the aggregate production planning strategies for the applications of SCM to the hospital. The strategies can be selected to apply the SCM to the hospital in the consideration of hospitals' PSL and total cost.

The study attempted to construct a HSCM model for drug delivery to avoid the drug shortage and to explore the relationship among the cost management, purchasing strategy, the logistic system, and e-health purchasing system. The concept of HSCM is based on the concept that when the actual demand occurs, the adjustable strategies for the dispatched quantity from different pharmaceutical companies will be computed to adjust the shortage demand. To achieve the optimal overall performance of the HSCM, it is necessary to pay attention to the variations in different ordering quantities and dispatched quantities of the required product when the hospital has the instability of the demand forecasting. The variations of the major parameters will be taken into consideration in order to determine the optimal supply chain's overall performance.

Furthermore, to explore the computational technology, Liao et al. [18] made a research on the optimal parameter settings, in which the regression models were developed and the genetic algorithm (GA) was applied to find out the optimal parameter settings for the HSCM. Vera Candioti et al. [19] explored the experimental design and multiple response optimization and found that the response surface methodology and the neural network (NN) are suggested to set the relationship between the factors and performances. This study used the simulation method to simulate the HSCM's dynamic character. The important factors and performances affecting HSCM were analyzed, and a simulation and NNs were used to identify the relationship between the HSCM factor levels and their performances. The objective of the study was to establish a robust HSCM with which to create adjustable ordering strategies.  Section 2 will present the scenario and the factor level settings in the HSCM, and Section 3 will present the conclusions.

## 2. The Scenario and Factor Level Settings in the HSCM

In this section, the HSCM and its factor level settings will be defined. In the framework of the HSCM, medical (product) purchase demands were compiled by the e-health purchasing system. Three pharmaceutical companies were contracted to supply three different drugs (drug A, drug B, and drug C). The e-health purchasing system included purchasing and coordination distribution mechanisms to compute and suggest the most robust HSCM factor level settings. The three drugs had the same indications and thus could be used to replace one another if there were a shortage. Orders for these drugs were processed in the following way: the hospitals sent their requests for each kind of drug to the e-health purchasing system, which collated the requests, computed the total quantities to be ordered, and submitted them to the pharmaceutical companies via the Internet. The pharmaceutical companies then delivered the requested quantities of the drugs ordered to the hospital. In order to circumvent the seasonal variation that affects actual demand, the pharmaceutical companies would maintain a safety stock of drugs at the warehouse's request. The warehouse would then take any inventory shortages into consideration when filling the hospital's orders. Small lot sizes and multiple deliveries could be implemented in order to save storage space.

Some research on HSCM performance has taken the total system cost into consideration [10–13]. In addition, patient safety is crucial to the quality of patient care and patient satisfaction [14–17]. Therefore, to explore the HSCM's performances, both the total system cost and patient safety should be simultaneously taken into consideration in the study. Why the patient safety is emphasized here is because, due to drug patent protection, medicine suppliers are market monopolies, which in some circumstances would lead to medicine shortage and hence endanger patient safety. Therefore, while exploring the patient safety for HSCM, the prevention of medicine should also be investigated. In case that there is any disruption in material flow of any medicine supplier, medicine shortage may occur, later causing medicine scarcity in some hospitals and hence resulting in a threat to patient safety [14–17, 20, 21]. Viewing that, patient safety is also taken into consideration for HSCM performance in the study.

In order to make the design of the HSCM as robust as possible, four steps were proposed.

**Step 1 (take the HSCM factors and their levels into consideration).**

In this step, in order to arrive at a robust design for the HSCM, the following important factors and their settings were considered. First, the noise and control factors were identified. In general, demand variability is difficult to control, and decision-makers always use the forecasting model to predict the quantities of the drugs that will be needed. The noise factor was identified as the demand variability, calculated using the normal probability distribution, *N*(*μ*, *σ* ^2^), in which *μ* was the mean (units/month) and *σ* ^2^ was the variance (units^2^/month^2^). In this study, the actual demand for the period from January to April was calculated as *N*(800,100^2^) and for the period from May to December as *N*(300,250^2^). The different calculations of demand variability highlighted the change in demand in different seasons.

The control factors were the safety stock (level 1: 400 units, level 2: 500 units, and level 3: 600 units), the maximum inventory (level 1: 2,000 units, level 2: 2,500 units, and level 3: 3,000 units), the reliability of the HSCM (level 1: 99 percent, level 2: 97 percent, and level 3: 96 percent), and the transportation capacity (level 1: 250 units, level 2: 500 units, and level 3: 1,000 units).

**Step 2 (identify the relationship between the factor levels and performance).**

To explore the relationship between the factor levels and performance, the mathematical function of the HSCM performance was discussed. Generally, there are two indexes of HSCM performance: the TSC and the PSL. In this study, the TSC included the costs of purchasing, inventory, transportation, and disposal. Equation (1) shows the TSC function:

(1) TSC=∑m=13∑t=112Pit∗Qit+Imt∗Pmt∗ht+QmtNm∗qmt+Omt∗dmt,

where *P*_*mt*_ is the price of drug *m* for the *t*th month, *m* = 1,2, 3, *t* = 1,2,…, 12; *Q*_*mt*_ is the quantity of drug *m* ordered for the *t*th month, *m* = 1,2, 3, *t* = 1,2,…, 12; *I*_*mt*_ is the inventory level of drug *m* for the *t*th month, *m* = 1,2, 3, *t* = 1,2,…, 12; *h*_*t*_ is the rate of storage cost per unit for the *t*th month, *t* = 1,2,…, 12; ⌈*Q*_*mt*_/*N*_*m*_⌉ is the ceiling function to calculate the number of deliveries of drug *m* for the *t*th month, *m* = 1,2, 3, *t* = 1,2,…, 12; *N*_*m*_ is the transportation capacity of drug *m* for each vehicle; *q*_*mt*_ is the transportation cost of drug *m* for the *t*th month, *m* = 1,2, 3, *t* = 1,2,…, 12; *O*_*mt*_ is the quantities of expired drug *m* for the *t*th month, *m* = 1,2, 3, *t* = 1,2,…, 12; and *d*_*mt*_ is the disposal cost per unit for drug *m* for the *t*th month, *m* = 1,2, 3, *t* = 1,2,…, 12.

The TSC is subject to

(2) Imt−1+Qmt−Dmt=Imt−SmtQmt=0,Qmin≤It−1≤QmaxQmax−It−1,It−1>QminIt=∑m=13Imt,

where *D*_*mt*_ is the actual demand for drug *m* for the *t*th month, *m* = 1,2, 3, *t* = 1,2,…, 12; *S*_*mt*_ is the shortage level of drug *m* for the *t*th month, *m* = 1,2, 3, *t* = 1,2,…, 12; *Q*_min_ is the maximum inventory level; *Q*_max_ is the minimum inventory level; and *I*_*t*_ is the total drug inventory level for the *t*th month, *t* = 1, 2,…, 12.

The PSL was also explored here. The service level was included in the PSL because shortage of drugs would cause harm to patients [14–17]. The PSL was defined as

(3) $$\begin{array}{c} PSL=1-\frac{\sum_{m=1}^{3}\sum_{t=1}^{12}S_{mt}}{\sum_{m=1}^{3}\sum_{t=1}^{12}D_{mt}}. \end{array}$$

Furthermore, the TSC and the PSL give rise to a tradeoff effect because, in ordering larger quantities, the PSL increases due to a decrease in the probability of an inventory shortage; however, increasing the inventory would increase the TSC. On the other hand, ordering smaller quantities would decrease the PSL because it would heighten the probability of an inventory shortage, which would cause a decrease of the TSC. Hence, the TSC and PSL are integrated into one function to aggregate multiple performances (MP). The MP is based on the concept of desirability function. Harrington [22] proposed the desirability function in 1965 as a criterion for response optimization. In 1980, Derringer and Suich [23] modified the function to make it more flexible in practical applications in order to serve as useful tool to solve a multiperformance problem. Derringer and Suich [23] defined the multiperformance as *D* = (∏_1_^*r*^ *d*_1_ *∗d*_2_ *∗* ⋯ *∗d*_*r*_)^1/*r*^, where *r* denoted performances. *d*_*i*_ denoted the desirability function of *i*th performance. Therefore, MP denoted the geometric mean of the *r* desirability values.

**Step 3 (obtain the optimal combinations of factor levels).**

The parameter settings for the simulation are summarized in Table 1. A total of 81 combinations (3*∗*3*∗*3*∗*3) for different control factor levels were simulated. The NN was applied here to construct the black-box mathematical functions. The input variables were the factors; the output variables were the normalized TSC (NTSC), the normalized PSL (NPSL), and the MP. The normalized mean of the TSC for each combination (TSC_mean_) was defined as NTSC = TSC_min_/TSC_mean_, where the TSC_min_ was the minimum TSC_mean_. Thus, the larger the NTSC the better, and the value was in the range of 0~1. The normalized mean of the PSL for each PSL_mean_ was defined as NPSL = PSL_mean_/PSL_max_, where the PSL_max_ was the maximum PSL_mean_. Thus, the larger the NPSL the better, and the value was in the range of 0~1. The MP was defined by the desirability function: $MP=\sqrt{NTSC*NPSL}$. The larger the MP the better, and the value was in the range of 0~1. The MP is expected to be maximized.

Three NN models (the MP model, the NTSC model, and the NPSL model) were obtained. In selecting the three optimal models, the lowest RMSE (the root mean-square error) was taken into consideration. The MP model in Table 2 and the NTSC model in Table 3 have a 4-3-1 (input nodes-hidden nodes-output node) structure, and the NPSL model in Table 4 has a 4-4-1 structure. The GA was used to find the optimal factor level combinations. The operational conditions of the GA were set as follows: the number of generations was 1,500, the population size was 100, the crossover rate was 0.85, and the mutation rate was 0.082. Based on the MP model, the results showed that the safety stock was at level 1.2 (420 units), the maximum inventory was at level 1.4 (2,200 units), the reliability of HSCM was at 99 percent, and the transportation capacity was at level 3 (1000 units). The MP value was 0.980. At this factor level combination, the NTSC was 0.890 and the NPSL was 0.875. To convert the NTSC into the TSC_mean_, the TSC_mean_ had to be 114,168. To convert the NPSL into the PSL_mean_, the PSL_mean_ had to be 0.921.

To complete the sensitivity analysis in order to discuss the changes in the optimal factor levels based on the optimal factor level combinations, Tables 5, 6, 7, and 8 show the changes in the NTSC, the TSC, and the PSL when one factor level was changed while the others were fixed. The results were as follows:

(1) When the safety stock level was increased from 1.2 to 3, the MP decreased from 0.980 to 0.760, the TSC_mean_ increased from 114,168 to 147,217, and the PSL_mean_ decreased from 0.921 to 0.714.
(2) When the maximum inventory level was increased from 1.4 to 3, the MP decreased from 0.980 to 0.843, the TSC_mean_ increased from 114,168 to 132,722, and the PSL_mean_ decreased from 0.921 to 0.792.
(3) When the reliability of the HSCM was increased from 1 to 3, the MP decreased from 0.980 to 0.857, the TSC_mean_ increased from 114,168 to 124,218, and the PSL_mean_ decreased from 0.921 to 0.805.
(4) When the transportation capacity was decreased from 3 to 1, the MP decreased from 0.980 to 0.900, the TSC_mean_ increased from 114,168 to 130,254, and the PSL_mean_ decreased from 0.921 to 0.846.

**Step 4 (discuss the available adjustable ordering strategies when there is a shortage of one of the three drugs).**

To explore the adjustable ordering strategies for drugs A, B, and C, Table 9 shows the optimal factor level combinations and their respective MP when one of them is subject to a shortage. When there is a shortage of drug A, B, or C, the following adjustable ordering strategies should be discussed:

(1) When there is a shortage of drug C but drugs A and B can be ordered, the MP would be 0.912, the factor level combination would be adjusted to a safety stock of 430 units, the maximum inventory level would be 2,300 units, the HSCM reliability would be 99 percent, and the transportation capacity would be 250 units. If the factor level combination cannot be adjusted, the MP would decrease to 0.825.
(2) When there is a shortage of drug B but drugs A and C can be ordered, the MP would be 0.924, the factor level combination would be adjusted to a safety stock of 420 units, the maximum inventory level would be 2,100 units, the HSCM reliability would be 99 percent, and the transportation capacity would be 1,000 units. If the factor level combination cannot be adjusted, the MP would decrease to 0.814.
(3) When there is a shortage of drug A but drugs B and C can be ordered, the MP would be 0.990, the factor level combination would be adjusted to a safety stock of 580 units, the maximum inventory level would be 2,800 units, the HSCM reliability would be 99 percent, and the transportation capacity would be 1,000 units. If the control factor level combination cannot be adjusted, the MP would decrease to 0.889.

## 3. Conclusions

In this paper, the authors explored adjustable ordering strategies for dealing with drug shortages. The HSCM simulation model, which could handle three drugs from the same class and with the same indications, was used to find the optimal robust design and adjustable ordering strategies to cope with a drug shortage, according to the hospital's needs and with the support of a purchasing alliance. Four steps were applied here to construct the HSCM model: the HSCM factors and their levels were taken into consideration; the relationships between the factor levels were identified; the optimal combinations of factor levels were obtained; and finally the adjustable ordering strategies to use when there was a shortage of one of the three drugs were discussed. The most significant contribution of this study is that it takes the adjustable ordering strategies of the HSCM into consideration when dealing with practical problems. The concept of adjustable ordering strategies will play an important role in future research on HSCMs. Also, some other contributions in the proposed computational technology are presented below:

1. The study used the NN to effectively deal with complex nonlinear relationship between the factor levels and performance. In addition, GA was used to obtain the optimal combination of factor levels from the NN, from any values within the upper and lower bounds for control factors.
2. Different settings of the same factor could be optimal for different performances. In the study, the performances of TSC and PSL are usually in conflict. A common method used for tackling the multiple performances problem is to give a weighted value to each performance, which needs to be judged and decided by a human subject. However, the desirability function proposed in the study does not need any human judgment. Therefore, it could be an attractive method in simplifying multiple performance problems because it forthright employs the upper and lower bound of each performance, without any human judgment. Also, the value of composite desirability MP derived in the study was 0.980, a value very close to 1. Hence, the desirability function in this study's NN model for the optimization of multiresponse problems can be a very useful tool to predict surface roughness.
3. In the study, the proposed methods, experimental design, NN, and GA, can be used for analysis, modeling, and optimization. Consequently, they can be applied in concurrent design process in HSCM.

The limitation of the study is that the control factors' levels are based on the preference of and the control of HSCM decision-makers. However, the noise factor's levels are in the control of external environment. Hence, while adjusting the optimal combination of factor levels, the designer must take the variations of the external environment into consideration.

## Figures and Tables

**Table 1:** The parameter settings for the HSCM simulation.

*P*_1*t*_: 29~37	*h*_*t*_: 0.25~0.3	*d*_1*t*_: 10~12	*q*_1*t*_: 7260~7676

*P*_2*t*_: 36~42		*d*_2*t*_: 11~13	*q*_2*t*_: 15250~16059

*P*_3*t*_: 48~56		*d*_3*t*_: 13~15	*q*_3*t*_: 33655~33586

**Table 2:** The neural networks for the MP model.

Structures (input nodes-hidden nodes-output nodes)	RMSE	
	Training	Testing
4-6-1	0.02982	0.02634
4-5-1	0.02632	0.02471
4-4-1	0.02444	0.02170
***4-3-1***	***0.02321***	***0.02018***
4-2-1	0.02651	0.02485
4-1-1	0.03145	0.03059

***Note***. The learning rate was set to autoadjust to value between 0.01 and 0.3; the momentum coefficient is 0.80; and the number of iterations is 15,000.

**Table 3:** The neural networks for the NTSC model.

Structures (input nodes-hidden nodes-output nodes)	RMSE	
	Training	Testing
4-6-1	0.03116	0.02824
4-5-1	0.02998	0.02755
4-4-1	0.02633	0.02463
***4-3-1***	***0.02254***	***0.02092***
4-2-1	0.02761	0.02537
4-1-1	0.02993	0.02495

***Note***. The learning rate was set to autoadjust to value between 0.01 and 0.3; the momentum coefficient is 0.80; and the number of iterations is 15,000.

**Table 4:** The neural networks for the NPSL model.

Structures (input nodes-hidden nodes-output nodes)	RMSE	
	Training	Testing
4-6-1	0.03136	0.02888
4-5-1	0.02872	0.02564
***4-4-1***	***0.02521***	***0.02338***
4-3-1	0.02766	0.02462
4-2-1	0.02899	0.02317
4-1-1	0.03100	0.02891

***Note***. The learning rate was set to autoadjust to value between 0.01 and 0.3; the momentum coefficient is 0.80; and the number of iterations is 15,000.

**Table 5:** The values for the MP, the TSC_mean_, and the PSL_mean_ resulting from a 20 percent change in the safety stock level.

MP	0.930	**0.980 **	0.925	0.900	0.880	0.870	0.848	0.830	0.810	0.780	0.760

Safety stock	1	**1.2**	1.4	1.6	1.8	2	2.2	2.4	2.6	2.8	3

TSC_mean_	121106	**114168 **	121956	124446	127345	129623	132539	134661	138439	144482	147217

PSL_mean_	0.874	**0.921**	0.869	0.846	0.827	0.817	0.797	0.780	0.761	0.733	0.714

**Table 6:** The values for the MP, the TSC_mean_, and the PSL_mean_ resulting from a 20 percent change in the maximum inventory level.

MP	0.910	0.930	**0.980 **	0.910	0.900	0.891	0.881	0.872	0.861	0.852	0.843

Maximum inventory	1	1.2	**1.4**	1.6	1.8	2	2.2	2.4	2.6	2.8	3

TSC_mean_	123050	120426	**114168 **	122851	124226	125583	126966	128418	129747	131421	132722

PSL_mean_	0.855	0.874	**0.921**	0.855	0.846	0.837	0.828	0.819	0.809	0.801	0.792

**Table 7:** The values for the MP, the TSC_mean_, and the PSL_mean_ resulting from a change in the reliability of the HSCM.

MP	**0.980 **	0.818	0.857

HSCM reliability	**1 **	2	3

TSC_mean_	**114168**	122850	124218

PSL_mean_	**0.921**	0.868	0.805

**Table 8:** The values for the MP, the TSC_mean_, and the PSL_mean_ resulting from a change in the transportation capacity.

MP	**0.980 **	0.910	0.900

Transportation capacity	**3 **	2	1

TSC_mean_	**114168**	136878	130254

PSL_mean_	**0.921**	0.855	0.846

**Table 9:** The control factor level combinations and their MPs when there is a shortage of drug A, B, or C.

Drugs to be ordered	Safety stock	Maximum inventory level	HSCM reliability	Transportation capacity	MP
A, B	430	2300	99%	250	0.912
A, C	420	2100	99%	1000	0.924
B, C	580	2800	99%	1000	0.990
